# Detection and Analysis of Syntenic Quantitative Trait Loci Controlling Cell Wall Quality in Angiosperms

**DOI:** 10.3389/fpls.2022.855093

**Published:** 2022-03-03

**Authors:** Francesco Pancaldi, Dennis Vlegels, Hugo Rijken, Eibertus N. van Loo, Luisa M. Trindade

**Affiliations:** Plant Breeding, Wageningen University & Research, Wageningen, Netherlands

**Keywords:** QTLs, syntenic QTLs, cell wall, synteny, biomass quality, orphan crops, crop improvement, breeding tools

## Abstract

Translational genomics can enable a quicker improvement of orphan crops toward novel agricultural applications, including the advancement of orphan biomass species for cultivation on marginal lands. In this sense, cell wall quality is a preeminent breeding target. However, tools to efficiently project genetic data on target traits across large sets of species are currently missing. This study aimed at closing this gap by developing a strategy to project a set of cell wall QTLs across a large group of plants by using genome synteny. This strategy is suited for large-scale analyses and detected 362 syntenic cell wall QTLs (SQTLs) across 74 angiosperms, including several (orphan) biomass species. SQTLs analyses revealed that they span large portions of the initial cell wall QTLs and are extensively conserved across diverse species. Moreover, numerous QTLs cell wall genes were conserved through SQTLs, including genes displaying allelic variation associated with cell wall composition. Functional analyses showed that highly conserved genes of SQTLs include important cell wall transcription factors and genes involved in the remodeling of cell wall polymers. For some of these gene families, SQTLs indicated the presence of differentially conserved genomic contexts for different gene members, highlighting their utility as a tool to pinpoint gene targets that maximize the likelihood of functional gene conservation. Overall, the results of this study can facilitate “universal” approaches for breeding (orphan) biomass crops, while the strategy for QTLs translation can be applied to other sets of traits and species, helping to unlock the potential of orphan species.

## Introduction

A key challenge faced by modern plant breeding is the effective valorization of the genetic resources of orphan crops (Kamei et al., 2016). These crops are currently underutilized in agriculture, despite being relevant for the subsistence of many local and regional communities, being promising for emerging agricultural markets, and carrying valuable traits (Kamei et al., 2016; Tadele, 2019). Orphan crops include plants that can potentially fit different agricultural applications, from diverse food species (cereals, legumes, vegetables, and tubers) to several industrial crops (oilseeds and biomass species, as well as plants suitable for multipurpose biorefineries) (see Tadele (2019) and Pancaldi and Trindade (2020) for an extensive list of orphan species). Moreover, many orphan crops are native of areas that face adverse climatic conditions (Pancaldi and Trindade, 2020). Therefore, their advancement could contribute to diversify agricultural markets, improve food diets, as well as mine useful traits to adapt staple crops to climate change (Kamei et al., 2016; Tadele, 2019). One of the main reasons for the underutilization of orphan crops in agriculture is the scarcity of genetic tools available for their improvement (Pancaldi and Trindade, 2020). This aspect, together with the niche markets currently shared by most of these crops, hampers their effective utilization for the purposes just discussed.

Several studies identified a turning point for the effective valorization of orphan crops in the rapid advancement of genome sequencing technologies, together with the dropping of their costs (Salentijn et al., 2007; Kang et al., 2016). In fact, *de novo* genome assemblies do not represent a constraint to plant research anymore, and the increasing availability of these resources for new species calls for approaches where they can be effectively used for transferring knowledge on traits of interest from model species to new crops (Salentijn et al., 2007; Pancaldi and Trindade, 2020). These approaches can be broadly referred as “translational genomics,” and generally encompass the analysis of genetic information on traits of interest from multiple species and their inter-species projection through genomic analyses (Kang et al., 2016). Typically, genetic data on the determinants of traits of interest are available as lists of candidate genes detected through reverse genetics, or as markers delimiting quantitative trait loci (QTLs) mapped in forward genetic studies (Pflieger et al., 2001; Rafalski, 2010). In principle, if the physical genomic positions of target genetic elements are available, genes, markers and QTLs could be projected between species by using bioinformatic tools such as the analysis of gene synteny (i.e., the conservation of the type and order of genes across the genomes of different species) (Zhao and Schranz, 2019; Pancaldi and Trindade, 2020). However, because of the current lack of pipelines suited for whole-trait analyses on large sets of species, these methodologies have been hardly applied at a large-scale level so far (Pancaldi and Trindade, 2020).

Among all the sectors where orphan crops could provide precious resources to plant breeding and agriculture, the field of the bio-based economy and of biomass crops is certainly one whose market opportunities are expected to grow more rapidly over the next decades (Piotrowski et al., 2015). In this sense, it has been shown that biomass crops for bio-based applications could be extensively grown on marginal lands (i.e., lands not used by agriculture and of poor natural value) to avoid competition with food crops (Carlsson et al., 2017; Mehmood et al., 2017; Pancaldi and Trindade, 2020). However, this requires crop varieties able to produce large and high-quality yield under the suboptimal (a)biotic conditions of marginal lands (Dauber et al., 2012; Blanco-Canqui, 2016). Interestingly, several perennial biomass species are native of marginal areas, and are naturally able to withstand the stresses that can be encountered there (Carlsson et al., 2017; Mehmood et al., 2017). However, the amount and quality of their biomass yield is not optimal, as most of these crops lay in an orphan state and never underwent breeding so far (Pancaldi and Trindade, 2020). Examples of these crops include miscanthus, switchgrass, poplar or willow, which share a sequenced genome; but also herbaceous and woody biomass species for which genetic resources are even more scarce, as giant reed, reed canary grass, black locust, and siberian elm (Pancaldi and Trindade, 2020). These crops should therefore be improved, and a preeminent target trait is certainly cell wall quality, which is the major determinant of biomass quality for bio-based applications (Van Der Weijde et al., 2013; Isikgor and Becer, 2015; Van Der Cruijsen et al., 2021). Specifically, the total content of the major cell wall components – cellulose, hemicelluloses, and lignin – as well as cellulose cristallinity, degree and type of hemicellulose substitutions, and the monolignol composition of the lignin polymers are all target characters at the basis of biomass quality (Van Der Weijde et al., 2013; Van Der Cruijsen et al., 2021). Applying translational genomics to the improvement of these traits in orphan biomass crops is expected to significantly speed up the development of varieties that combine resilience to marginal land conditions with production of large and good-quality biomass yields (Pancaldi and Trindade, 2020).

Within the context above, this study aimed at setting up a strategy for the inter-species projection of a set of 610 QTLs controlling cell wall quality previously mapped in 8 diverse plant species (detailed information in Supplementary Table 1) across a wide group of angiosperms, by using genome synteny. In this strategy, cutting-edge bioinformatic tools for network synteny analysis in large sets of genomes were applied to infer the syntenic conservation of the QTLs across all the plants of the study, leading to the detection of numerous conserved syntenic QTL regions. Syntenic cell wall QTLs were then characterized in terms of extensiveness among plants, fragmentation of their syntenic conservation, and conserved candidate genes. This allowed to make general inferences on the functional relevance of our translational genomics approach, to improve our knowledge on the conservation of critical genes at the basis of plant cell walls, and to highlight important candidate genes for further studies in (orphan) plant species. The strategy developed in this study can be applied to other traits, as well as other sets of QTLs and species, representing a novel tool to assist breeding research in orphan crops.

## Materials and Methods

### Collection of Cell Wall Quantitative Trait Loci

Scientific literature was searched for all the QTLs related to cell wall quality traits mapped in diverse species and delimited by molecular markers whose physical genomic position was reported or could be retrieved by BLAST (Altschul et al., 1990) or regression of genetic to physical genomic maps. This search was made in August 2019 and retrieved 610 QTLs for different traits related to cell wall quality from 19 different publications and 8 diverse plant species (*Arabidopsis thaliana*, *Eucalyptus grandis*, *Glycine max*, *Miscanthus sinensis*, *Oryza sativa*, *Populus trichocarpa*, *Sorghum bicolor*, and *Zea mays*) (see Supplementary Table 1 for the full list of QTLs, traits, references, and QTL positions in the genome of each species).

### Collection of Plant Genomes

All the angiosperm genomes sequenced and published by the end of 2018 and available with at least a scaffold-level assembly were searched for in several online databases. For each genome, a BED file indicating gene positions and a FASTA file reporting protein sequences coded by all the annotated genes were retrieved. Genomes were checked for assembly completeness by using the BUSCO Viridiplantae gene set (Seppey et al., 2019) and for assembly fragmentation by assessing the number of scaffolds and the N50 statistics. To select reliable genome assemblies for synteny analysis, genomes with <75% BUSCO genes and <10 genes per scaffold on average have been excluded from the collected set. Through these criteria, a total of 151 genomes from 134 species were collected and used in all further analyses (Supplementary Table 2).

### Identification of Candidate Cell Wall Genes in All the Genomes Used

Scientific literature was searched for all the genes known to play a role in plant cell wall synthesis and functioning (Supplementary Table 3). As the vast majority of cell wall genes turned out to be discovered or studied in *Arabidopsis thaliana*, the proteome of this species was downloaded from UniProt (UP000006548) (Uniprot Consortium, 2018) and filtered for the identified cell wall functions based on UniProt (Uniprot Consortium, 2018), TAIR (Berardini et al., 2015), and NCBI (NCBI Resource Coordinators, 2018) protein annotations. This filtering led to a list of 1311 Arabidopsis cell wall proteins, which were then extracted from the PEP file of the Arabidopsis genome and annotated for their domain architecture using HMMER (default parameters) (Wheeler and Eddy, 2013) and all the HMM alignments of the PFAM database (Finn et al., 2014). The 1311 Arabidopsis cell wall proteins were then used in a search for homologs based on both BLAST (Evalue = 1E–3) and HMMER (for matching PFAM protein architecture; default parameters) across the 151 genomes of the study. The set of candidate cell wall proteins identified was further adjusted by an iterative search of cell wall gene homologs based on BLAST and HMMER with the use of the identified candidate cell wall proteins as queries. In addition, known cell wall genes specific to certain species were used separately from the set of 1311 Arabidopsis cell wall genes as queries for homologs search as performed with the Arabidopsis genes. All together, these searches yielded 252471 candidate cell wall genes with functional annotation across the 151 genomes of the study (Supplementary Table 4).

### Construction of the Cell Wall Quantitative Trait Loci Synteny Network

Genome synteny was analyzed across all the 151 genomes of the study by following the methodology developed by Zhao and Schranz (2017) for large-scale network synteny analysis. In brief, Diamond (Buchfink et al., 2015) was used to perform BLAST-like alignments of all the proteins of each genome against all the other proteins of that genome and all the proteins of every other genome (Evalue = 1E–3). In this way, homologous genes between different species were identified across all pairs of genomes. Subsequently, MCScanX (Wang et al., 2012) was used to detect synteny (i.e., conserved gene order across multiple genomes) by evaluating the positions of the homologous genes from each genome comparison. MCScanX was run with default parameters except -s (number of colinear genes to claim a syntenic block) set to 3. The outputs of MCScanX were organized in a synteny network, in which each node is a gene and edges represent syntenic connections between genes. The synteny network was then filtered to retain only pairs of nodes in which at least one of the genes was included in the initial 610 cell wall QTLs (R, custom script). The output of this filtering was in turn further subset as described in section “Filtering the Syntenic Quantitative Trait Loci Network” and constitutes the network of syntenic relationships of all the genes included in the 610 cell wall QTLs across all the 151 genomes of the study (Supplementary Table 5).

### Filtering the Syntenic Quantitative Trait Loci Network

To identify conserved syntenic QTL regions representing reliable conservation of the initial 610 cell wall QTLs, it was assessed with which plant families the QTLs of each initial species displayed the highest synteny levels (calculated as the average percentage of genes of each initial QTL syntenic to the genomes of each plant family). The distributions of synteny levels were plotted in separate boxplots for each species/family for which cell wall QTLs were retrieved (Supplementary Figure 1). These boxplots were then used to filter the QTL synteny network to retain only the syntenic connections between genes of the initial QTLs and other genes belonging to genomes included in the upper quartile of each boxplot distribution (R, custom script). Table 1 illustrates the groups of families selected for each species for which initial cell wall QTLs were available. As a next step, the fragmentation of QTL synteny across the genomes included in each group of Table 1 was also assessed, to discriminate between cases in which small QTL fragments were syntenic toward several different genomic regions of a target species and cases in which large QTL(s) segment(s) were syntenic toward a single region in a target species (implying higher likelihood of QTLs functional conservation). To this aim, the synteny level of each initial QTL against each of the chromosomes of the species included in the groups of Table 1 was assessed, and the syntenic QTL network was filtered to retain only syntenic connections between QTL genes and other genes located on chromosomes on which at least 50% of QTL’s genes showed synteny. The filtered syntenic QTL network is included in Supplementary Table 5 and contains 494026 genes (nodes) from 87 genomes.

**TABLE 1:** Lists of plant families used for SQTLs detection for each of the plant families for which initial QTLs were retrieved.

Initial QTLs families	Plant families selected for SQTL detection
Brassicaceae	Brassicaceae, Malvaceae, Cleomaceae, Anacardaceae, Actinidiaceae, Myrtaceae
Fabaceae	Fabaceae, Salicaceae, Moraceae, Rhamnaceae, Linaceae, Cannabaceae, Euphorbiaceae, Rosaceae, Vitaceae, Cucurbitaceae, Crassulaceae, Nelumbonaceae, Ranunculaceae, Myrtaceae, Papaveraceae, Lythraceae, Anacardaceae, Rutaceae, Malvaceae, Brassicaceae, Cleomaceae, Amaranthaceae, Actinidiaceae, Convolvulaceae, Solanaceae, Rubiaceae, Oleaceae, Pedaliaceae, Phrymaceae, Asteraceae, Apiaceae
Myrtaceae	Lythraceae, Anacardaceae, Rutaceae, Malvaceae, Cucurbitaceae, Rosaceae, Rhamnaceae, Fabaceae, Actinidiaceae, Salicaceae, Rubiaceae, Vitaceae, Oleaceae, Pedaliaceae, Phrymaceae, Apiaceae
Salicaceae	Salicaceae, Linaceae, Fabaceae, Euphorbiaceae, Moraceae, Rhamnaceae, Vitaceae, Cannabaceae, Rosaceae, Crassulaceae, Cucurbitaceae, Nelumbonaceae, Ranunculaceae, Papaveraceae, Myrtaceae, Lythraceae, Anacardaceae, Rutaceae, Malvaceae, Brassicaceae, Cleomaceae, Amaranthaceae, Lauraceae, Theaceae, Amborellaceae, Actinidiaceae, Convolvulaceae, Solanaceae, Rubiaceae, Oleaceae, Pedaliaceae, Phrymaceae, Asteraceae, Apiaceae
Poaceae	Arecaceae, Araceae, Bromeliaceae, Asparagaceae, Orchidaceae, Musaceae, Poaceae

*The selection of plant families was based on the synteny level of the different initial QTLs against the 151 species of the project, as described in paragraph “Filtering the Syntenic Quantitative Trait Loci Network”.*

### Identification of Syntenic Cell Wall Quantitative Trait Loci

The filtered syntenic QTL network was used to detect syntenic cell wall QTLs within each group of Table 1 by following a “double-clustering” approach. First, to identify sets of genes highly syntenic with each other and with initial cell wall QTL(s), the R igraph package was used to identify all the communities of at least 10 nodes within the QTL synteny network (Louvain algorithm; 16644 communities detected; modularity = 0.99). These communities turned out to typically include all the members of a single gene type that are syntenic across the genomes inspected. Therefore, to detect syntenic QTL *regions* from single-homologs syntenic communities, a second clustering was applied to the detected communities. In this step, the identifier(s) of the QTL(s) harbored by (some of) the genes included within each detected community were annotated to the communities themselves. The annotated communities were then used to calculate the all-vs-all similarity of the communities themselves based on the initial cell wall QTLs represented in each community (R, custom script; Jaccard similarity algorithm). Similarities between communities were saved into a network and their distribution was plotted in a boxplot. This network was then filtered to retain only the connections between communities supported by a similarity >0.6 (upper quartile of the distribution of similarities). In turn, the QTL synteny network was then also filtered to retain only syntenic relationships between genes included in the filtered communities. The genomic regions whose genes are included in the communities contained in the filtered QTL synteny network represent the syntenic cell wall QTLs (SQTLs).

### Analysis of Syntenic Cell Wall Quantitative Trait Loci

The SQTLs detected through the methodology above were analyzed in terms of extensiveness, fragmentation, frequency and size across species, as well as for the co-localization of functionally different initial QTLs. Moreover, the conservation of cell wall genes through syntenic cell wall QTLs was also analyzed. In this respect, all the analyses performed are described in the next sections of the manuscript, and were performed by using R, Excel, or SPSS.

## Results

### Preliminary Analysis of Cell Wall Quantitative Trait Loci and Cell Wall Gene Data

To develop an effective methodology for projecting cell wall QTLs across a wide set of plants with the use of gene synteny, 610 cell wall QTLs previously mapped in arabidopsis (Brassicaceae), soybean (Fabaceae), poplar (Salicaceae), eucalyptus (Myrtaceae), miscanthus, maize, sorghum, and rice (Poaceae) were collected from scientific literature (Section “Collection of Cell Wall Quantitative Trait Loci” and Supplementary Table 1). In addition, ∼250000 candidate cell wall genes were identified across >150 angiosperm genomes through a combined BLAST- (Altschul et al., 1990) and HMMER-based (Wheeler and Eddy, 2013) search on a large set of characterized cell wall genes retrieved from scientific literature (Section “Identification of Candidate Cell Wall Genes in All the Genomes Used” and Supplementary Table 4). To assess the feasibility of genomically translating the QTLs and the candidate cell wall genes therein across a wide set of species through gene synteny, QTLs and cell wall genes were initially assessed for QTL gene content, QTL length variability, and general synteny of both QTLs and candidate cell wall genes.

Since some of the 610 cell wall QTL intervals retrieved from scientific literature referred to genetic maps, the QTLs were first translated to physical genomic positions (Section “Collection of Cell Wall Quantitative Trait Loci” and Supplementary Table 1), and the gene content of the genomic QTL regions was analyzed. Knowing the gene content of target regions to be projected between species through the use of genome synteny is highly relevant, since synteny is defined at the gene level (i.e., conservation of the type and order of genes between species) (Zhao and Schranz, 2017). The analysis of QTLs gene content revealed that 16 QTLs are located on genomic regions without genes, 37 QTLs do not span any candidate cell wall gene, and 50 QTLs include only one candidate cell wall gene (Supplementary Table 1). The latter group raises a particular interest, as the candidate cell wall gene harbored by each of those QTLs may represent the causative gene of each QTL. Therefore, these genes were collected and analyzed, revealing that they vary considerably in terms of cell wall function(s) and process(es) in which they play a role (Supplementary Table 6).

By using the physical QTL ranges, the QTLs length variation was also assessed (meant as both nucleotide length of QTL ranges and total number of genes within QTLs). This is another important parameter to be considered in synteny analysis, since the length of target genomic regions for synteny analysis is known to potentially affect the fragmentation of the syntenic regions obtained (Liu et al., 2018). In this sense, ANOVA results indicated that QTLs collected for the Poaceae species and, to a lesser extent, for eucalyptus, span significantly longer nucleotide regions than the QTLs of arabidopsis, poplar, and soybean, whose length ranges do not differ significantly from each other (Bonferroni’s LSDs; α = 0.05). However, given the highly significant differences between the species for which QTLs were retrieved in terms of gene density of the QTL regions (*P* < 0.000), arabidopsis QTLs displayed a significantly higher gene content than the QTLs from all the other species (*P* < 0.000). In turn, QTLs from Poaceae, poplar, eucalyptus, and soybean did not differ from each other in terms of gene content, with the only exception of poplar and eucalyptus QTLs (Bonferroni’s LSDs; α = 0.05). The patterns observed for the overall QTLs gene content are similar to what observed for the QTLs cell wall gene content. Accordingly, the correlation between total QTL gene content and QTL cell wall gene content turned out to be particularly high (ρ = 0.91, *P* < 0.000).

**FIGURE 1:**
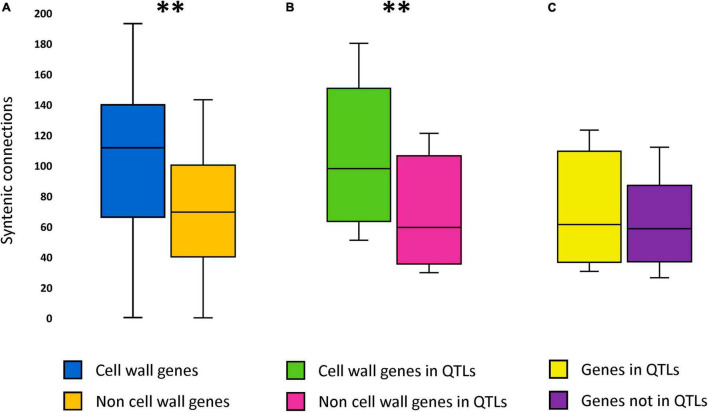
The level of synteny of different relevant classes of genes. **(A)** Boxplots of the number of syntenic connections of cell wall genes and of non-cell wall genes from the 151 genomes of the study. **(B)** Boxplots of the number of syntenic connections of cell wall genes and of non-cell wall genes from the 594 cell wall QTLs of the study spanning at least one gene. **(C)** Boxplots of the number of syntenic connections of cell wall QTL genes and of genes not included in cell wall QTLs from the 151 genomes of the study. **Significant at alpha = 0.01.

In addition to QTL gene content and QTL length variability, the general synteny of the candidate cell wall genes and of the 594 cell wall QTLs spanning at least one gene was also assessed, in order to estimate the overall feasibility of using gene synteny for inter-species QTLs projection. These analyses were performed by using the general synteny network of the 151 genomes of the study and the filtered QTL synteny network, respectively (see Section “Construction of the Cell Wall Quantitative Trait Loci Synteny Network” and “Filtering the Syntenic Quantitative Trait Loci Network”). Results revealed that candidate cell wall genes are significantly more syntenic than other genes not related with cell wall across all the genomes analyzed. This pattern holds true both when assessed across whole genomes and when assessed over cell wall QTL regions only. Specifically, *t*-tests showed that each of the 252471 candidate cell wall genes identified across the 151 angiosperm genomes of the study displays synteny with other 101 homologs in other species on average, compared to 68 average syntenic connections for the non-cell wall genes (*P* < 0.000) (Figure 1A). Within QTLs, these figures amount to 107 and 67 syntenic connections, respectively (*t*-test’s *P* < 0.000) (Figure 1B). To conclude, the synteny level of QTL genes (both cell wall and non-cell wall) does not significantly differ from the one of genes outside QTL regions (*t*-test; α = 0.05) (Figure 1C). Nevertheless, with an average of 69 syntenic connections per gene, QTL genes can overall be considered highly syntenic (Figure 1C).

### Detection and Descriptive Analysis of Syntenic Cell Wall Quantitative Trait Loci

**FIGURE 2:**
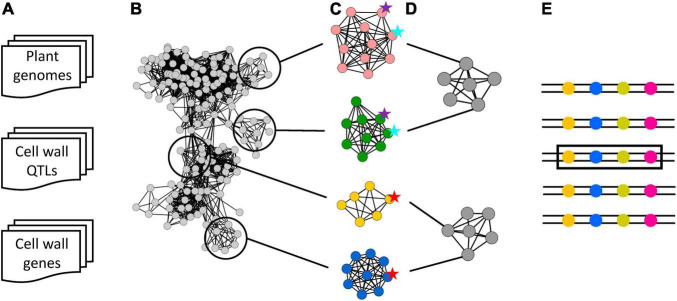
The pipeline followed for detecting syntenic cell wall QTLs (SQTLs). **(A)** The starting data sets of 151 angiosperm genomes, of 610 cell wall QTLs from 8 diverse plant species, and of ∼250000 candidate cell wall genes identified across all the genomes of the study. **(B)** A synteny network of QTL genes across the species of the study was created, where each node (gray circles) represents a gene and black edges represent syntenic connections between genes. **(C)** Syntenic communities grouping the members of specific gene families that are syntenic across (groups of) plants were detected and annotated for the initial cell wall QTLs harbored by (some of) their members. Colored circles represent genes, and circles with the same color are genes belonging to the same syntenic community. Colored stars represent initial cell wall QTLs, with different colors indicating different initial cell wall QTLs. **(D)** Syntenic communities were clustered into SQTLs based on similarity of the cell wall QTLs harbored by each community. A SQTLs network was therefore obtained, where each node represent a syntenic community (gray circles), black edges between communities indicate a similarity >0.6 between two syntenic communities in terms of cell wall QTLs harbored by them. **(E)** Representation of the genomic meaning of SQTLs. Each double strip with circles represents the genome of a species, and colored circles represent genes of different types (different colors). A SQTL represents a genomic region syntenic between multiple species (same genes in the same order) and that in at least one species spans at least (a part of) one initial cell wall QTL (black rectangle).

**FIGURE 3:**
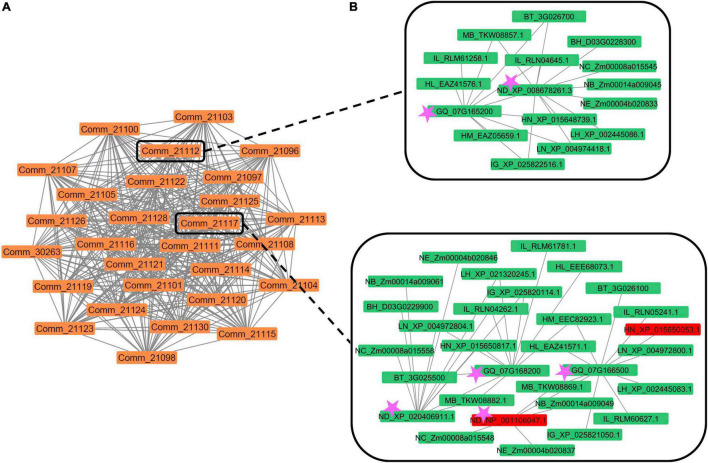
**(A)** Example of a SQTL (SQTL_196). The orange nodes represent all the colinear syntenic communities that group syntenic genes of which at least some are retained within initial cell wall QTLs. **(B)** Two of the syntenic communities that constitute the SQTL_196. Green rectangles represent community nodes. Pink stars indicate maize (ND_) and miscanthus (GQ_) nodes included within initial cell wall QTLs. Red nodes indicate a gene from maize (ND_NP_001106047.1) and a gene from rice (HN_XP_015650053.1) that correspond to the maize *Brown Midrib 3* locus and the rice *CAldOMT1* locus, respectively. Both these loci encode for a *COMT* gene involved in lignin synthesis and are known to display allelic variation associated to phenotypic variation in cell wall quality in across maize and rice populations (see Supplementary Table 9).

As previously mentioned, the main goal of this study was to develop a methodology to efficiently project an initial set of 610 cell wall QTLs across 151 angiosperm genomes by using gene synteny, to identify conserved syntenic cell wall QTLs (SQTLs). The high synteny of the cell wall QTLs collected from scientific literature and of the candidate cell wall genes therein (Section “Preliminary Analysis of Cell Wall Quantitative Trait Loci and Cell Wall Gene Data”) promised success for reaching this goal, and the approach described in Figure 2 and Sections “Construction of the Cell Wall Quantitative Trait Loci Synteny Network, Filtering the Syntenic Quantitative Trait Loci Network, and Identification of Syntenic Cell Wall Quantitative Trait Loci” was therefore designed. In this pipeline, the genes contained in the 594 cell wall QTLs spanning at least one gene were evaluated for syntenic conservation across all the genomes of the study by building a synteny network of QTL genes where each node represents a gene and edges connect syntenic genes (Figure 2B). The network was first used to evaluate the degree of synteny of each initial QTL across all the genomes of the study, leading to the identification of the groups of Table 1, which list species among which the synteny of the different initial QTLs is maximized, increasing the likelihood of QTLs’ functional conservation across species. In turn, each group of Table 1 was used to cluster the corresponding genes of the QTL synteny network first into single-locus communities (Figure 2C), and second into groups of communities representing syntenic regions sharing same initial QTLs (Figure 2D). These groups constitute the syntenic cell wall QTLs (SQTLs), which can be defined as genomic regions conserved across multiple species and spanning (a part of) one or more known cell wall QTLs in at least one species. Figure 3 shows a meaningful example of SQTL.

The pipeline above led to the detection of 362 SQTLs across the five groups of angiosperm genomes of Table 1 (see Supplementary Table 7 for a list of all the SQTLs and all their genes). These SQTLs span a total of 398231 genes (81% of the genes within the QTL synteny network) and of 74 different species (55% of the species used in the study). On average, each SQTL groups 1100 genes (CV = 152%) from 18 different species (CV = 37%) with a mean of 62 genes per species (CV = 169%). Of all the genes within the 362 SQTLs, 24987 are candidate cell wall genes (83% of the cell wall genes of the QTL synteny network), with an average of 69 cell wall genes per SQTL (CV = 160%) and of 4 cell wall genes per species within each SQTL (CV = 168%). Out of the 362 SQTLs, 92 do not contain any candidate cell wall gene. These 92 SQTLs contain significantly less genes and span significantly less species than the other SQTLs (*t*-test; *P* < 0.05 for both). To conclude, within the 270 SQTLs containing candidate cell wall genes, these represent on average 9% of the total SQTLs genes (CV = 85%, range = 1–63%).

To evaluate the validity of the approach followed for SQTLs detection, as well as to gain insights into the patterns of conservation of the cell wall QTLs across the 74 angiosperm species represented within SQTLs, the 362 SQTLs were detailly characterized for several attributes. These include the overall representation and fragmentation of initial cell wall QTLs across SQTLs, the frequency and extensiveness of SQTLs across relevant (groups of) plant species, the SQTL size (both overall and across distinct plants), and the general patterns of candidate cell wall gene conservation through SQTLs.

**FIGURE 4:**
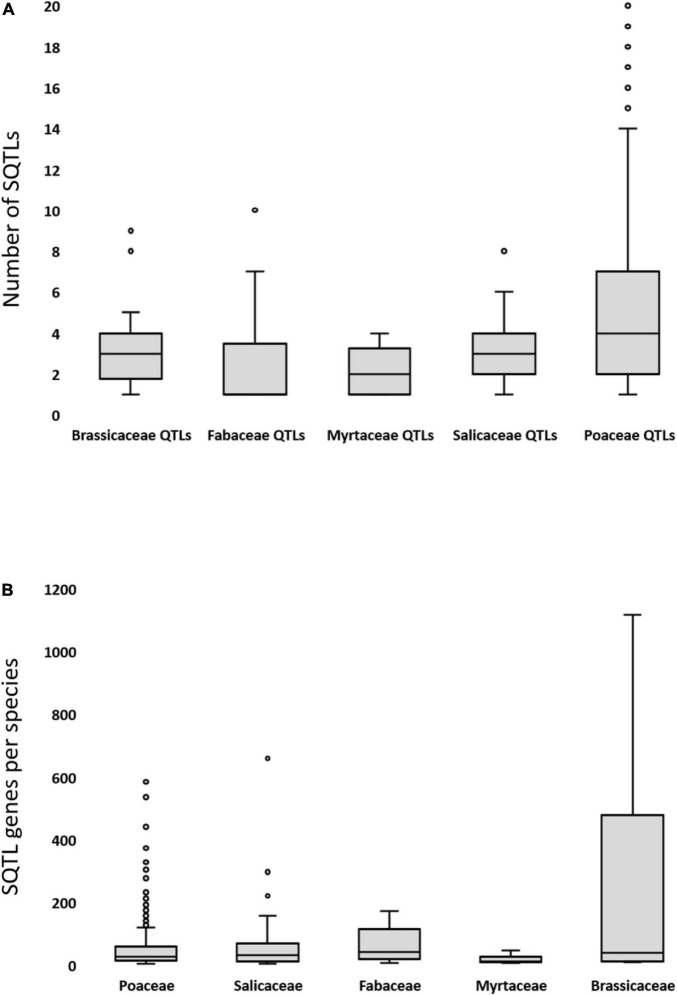
**(A)** The level of fragmentation of the initial cell wall QTLs from the different groups of Table 1 over the SQTLs in which they are involved. Fragmentation is expressed as number of different SQTLs over which different parts of initial QTL resulted involved. **(B)** The variability of SQTL length (expressed as number of SQTL gene per species involved in a SQTL) of the SQTLs detected for the plant groups of Table 1.

Regarding the representation and fragmentation of initial cell wall QTLs within SQTLs, our analyses revealed that 512 of the 594 initial cell wall QTLs spanning at least one gene are involved in SQTLs. Of these 512 QTLs, 90 are involved into one SQTL only, which includes 39% of the initial QTLs’ genes on average (range = 0.4–84%). The other 422 QTLs are instead involved into two or more SQTLs (average of 5; range = 2–31), with each SQTL spanning 13% of the initial QTLs’ genes on average (CV = 68%; range = 0.3–42%). QTL fragmentation over multiple SQTLs is not even across the five plant groups of Table 1. Specifically, while the QTLs from the dicot species are divided over 2.7 SQTLs on average, for the Poaceae QTLs this average is significantly higher (5.2 SQTLs; *t*-test’s *P* < 0.000), revealing a higher level of fragmentation (Figure 4A). In addition, the length of QTL fragments conserved through SQTLs is significantly shorter in Poaceae, Myrtaceae and Salicaceae (34, 11, 43 genes on average respectively) than in the Brassicaceae and Fabaceae groups (163 and 113 genes on average, respectively; ANOVA; *P* < 0.000). To conclude, the representation of initial cell wall QTLs within SQTLs was analyzed also from the point of view of SQTLs. This revealed that each of the 362 SQTLs spans 6 different initial cell wall QTLs (CV = 105%; range = 1–55) that come from 2 different initial QTL species (CV = 49%; range = 1–5) on average.

As previously mentioned, other analyses were performed to inspect the frequency and extensiveness of SQTLs across species. In this regard, SQTLs turned out to be numerous across all the 5 initial groups of Table 1, even if with substantial differences reflecting the asymmetry in the initial QTLs availability between plant groups and the intrinsic genomic relatedness of the species within each group. In fact, while 281 SQTLs were detected in the Poaceae (∼75% of the total; the group with the highest number of initial QTLs and a well-known example of highly syntenic plant family), the SQTLs detected across the other groups of Table 1 range from 6 for Myrtaceae to 50 for Salicaceae (Table 2). Despite the diversity in the absolute SQTL abundance across plant groups, SQTLs from all the groups are extensive across the species of each group, as each SQTL spans on average 54% of the species included in the group of Table 1 used for its detection. The extensiveness of SQTLs is again highest in the Poaceae group, where each SQTL spans on average 62% of the “Poaceae” species from Table 1 (Table 2). Nevertheless, the percentages found in the dicot groups are also relatively high (Table 2), considering the higher evolutionary diversity of these species compared to the Poaceae group.

**TABLE 2:** The extensiveness of SQTLs across the groups of species used for their detection.

SQTL group	Total species in SQTL group	Total number of SQTLs	Average species spanned by SQTLs	Percentage over total species
Brassicaceae	29	9	6	21%
Fabaceae	111	16	31	28%
Myrtaceae	59	6	21	35%
Salicaceae	112	50	25	22%
Poaceae	27	281	17	62%

Another parameter that was assessed is SQTLs size (meant as total number of genes spanned by SQTLs in each of the species included in SQTLs). Results revealed that SQTLs size is highest in SQTLs mapped within the Brassicaceae group (267 genes/species/SQTL), being significantly higher than in all the other groups of Table 1 (51 genes/species/SQTL as average across all other groups; ANOVA’s *P* < 0.000) (Figure 4B). Moreover, all the other groups were found to not significantly differ between each other in terms of SQTLs size (ANOVA’s α = 0.05). Interestingly, Brassicaceae is the group whose initial QTLs display the highest gene content (see Section “Preliminary Analysis of Cell Wall Quantitative Trait Loci and Cell Wall Gene Data”), and QTL gene content was positively correlated with SQTLs size across all initial QTLs species (ρ = 0.91, *P* < 0.000).

To conclude, the representation of candidate cell wall genes from the initial QTLs within the 362 SQTLs was also analyzed. This revealed that 28 of the 512 initial QTLs represented in SQTLs have their candidate cell wall genes not conserved at all through SQTLs. Interestingly, these 28 QTLs span significantly less genes and cell wall genes than the other QTLs (*t*-test, *P* < 0.05 for both). However, their nucleotide length does not significantly differ from the one of the other QTLs (*t*-test, α = 0.05). The 484 QTLs with candidate cell wall genes represented within SQTLs have instead on average 72% of their candidate cell wall genes conserved in SQTLs (CV = 37%; range = 2–100%). In conclusion, in terms of traits and functions of the candidate cell wall genes, chi-square tests revealed that the candidate cell wall genes conserved through SQTLs are significantly enriched in transcription factors (+1–2%) and significantly de-enriched in lignin genes (–4–6%; *P* < 0.000). Specifically, this holds true when the frequencies of candidate cell wall gene functions within SQTLs are compared with the corresponding frequencies in the full list of candidate cell wall genes, in the list of candidate cell wall genes within the QTL synteny network, and in the list of candidate cell wall genes within the 594 initial QTLs spanning at least one gene.

### Analysis of the Candidate Cell Wall Genes Within Syntenic Cell Wall Quantitative Trait Loci

The 362 SQTLs described in paragraph “Detection and Descriptive Analysis of Syntenic Cell Wall Quantitative Trait Loci” represent genomic regions that are syntenic across crops and that are known to display patterns of allelic variation associated with variation in cell wall composition in the species where they span known cell wall QTLs. The study of the candidate cell wall genes included in SQTLs could therefore contribute to identify relevant targets for crop improvement, to improve our knowledge on the degree of conservation of critical cell wall genes, and to design novel breeding strategies. In line with these goals, SQTLs were detailly functionally analyzed as described in the next sections.

#### Cell Wall Genes Within the Syntenic Cell Wall Quantitative Trait Loci With Highest Co-localization With Initial Quantitative Trait Loci

As previously mentioned, the 362 SQTLs were checked for extensiveness across both species and initial QTLs (section “Detection and Descriptive Analysis of Syntenic Cell Wall Quantitative Trait Loci”). By assessing these parameters, we identified the 22 SQTLs that fall in the upper quartile of SQTLs distribution for both number of initial cell wall QTLs and number of diverse plant species represented within SQTLs (Table 3). Because of these properties, these 22 SQTLs could likely represent relevant regions for conserved mechanisms of cell wall quality control across several species, and the types of candidate cell wall genes that they contain were analyzed.

**TABLE 3:** The 22 SQTLs selected for candidate gene inspection based on a high co-localization of initial QTLs and the inclusion of diverse plant species.

SQTL	SQTL group	Number of QTLs co-localizing in SQTL	Total species in SQTL	Species included in SQTL
SQTL_217	Fabaceae	6	2	Eucalyptus grandis; Glycine max
SQTL_23	Myrtaceae	9	2	Eucalyptus grandis; Populus trichocarpa
SQTL_121	Myrtaceae	7	3	Arabidopsis thaliana; Eucalyptus grandis; Populus trichocarpa
SQTL_169	Myrtaceae	7	4	Eucalyptus grandis; Glycine max; Populus trichocarpa; Populus trichocarpa
SQTL_187	Salicaceae	14	3	Arabidopsis thaliana; Eucalyptus grandis; Populus trichocarpa
SQTL_246	Salicaceae	11	3	Arabidopsis thaliana; Eucalyptus grandis; Populus trichocarpa
SQTL_50	Salicaceae	8	3	Arabidopsis thaliana; Eucalyptus grandis; Populus trichocarpa
SQTL_174	Salicaceae	8	3	Eucalyptus grandis; Populus trichocarpa; Populus trichocarpa
SQTL_14	Salicaceae	8	2	Arabidopsis thaliana; Populus trichocarpa
SQTL_39	Salicaceae	7	3	Arabidopsis thaliana; Eucalyptus grandis; Populus trichocarpa
SQTL_53	Salicaceae	6	4	Arabidopsis thaliana; Eucalyptus grandis; Populus trichocarpa; Populus trichocarpa
SQTL_2	Poaceae	45	4	Miscanthus sinensis; Oryza sativa; Sorghum bicolor; Zea mays
SQTL_160	Poaceae	25	4	Miscanthus sinensis; Oryza sativa; Sorghum bicolor; Zea mays
SQTL_188	Poaceae	24	4	Miscanthus sinensis; Oryza sativa; Sorghum bicolor; Zea mays
SQTL_245	Poaceae	23	4	Miscanthus sinensis; Oryza sativa; Sorghum bicolor; Zea mays
SQTL_20	Poaceae	20	2	Sorghum bicolor; Zea mays
SQTL_91	Poaceae	20	4	Miscanthus sinensis; Oryza sativa; Sorghum bicolor; Zea mays
SQTL_47	Poaceae	20	4	Miscanthus sinensis; Oryza sativa; Sorghum bicolor; Zea mays
SQTL_56	Poaceae	19	3	Oryza sativa; Sorghum bicolor; Zea mays
SQTL_69	Poaceae	18	2	Miscanthus sinensis; Zea mays
SQTL_73	Poaceae	16	4	Miscanthus sinensis; Oryza sativa; Sorghum bicolor; Zea mays
SQTL_60	Poaceae	16	3	Miscanthus sinensis; Sorghum bicolor; Zea mays

**FIGURE 5:**
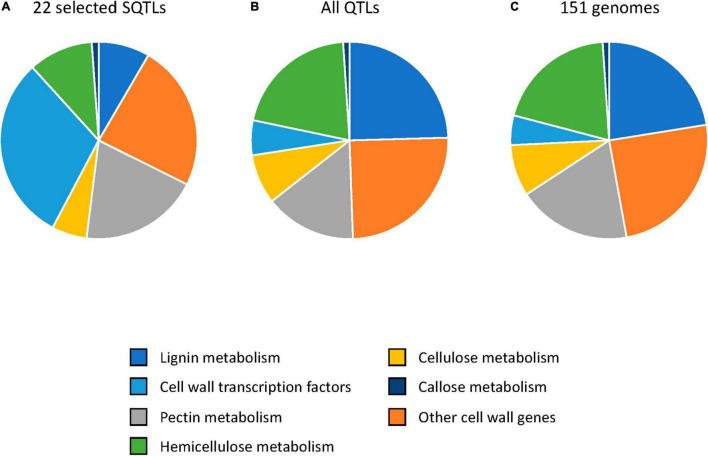
The proportions of cell wall genes participating to different cell wall processes observed among a set of 22 SQTLs showing an exceptionally high co-localization of initial cell wall QTLs from diverse plant species and highly conserved across different angiosperms **(A)**, among the genes included in all the initial 594 cell wall QTLs spanning at least one gene **(B)**, and among all the genes from the 151 angiosperm genomes of the study **(C)**.

In total, 1493 candidate cell wall genes were extracted from the 22 selected SQTLs (Supplementary Table 8). Notably, the proportions of candidate cell wall genes belonging to different cell wall processes observed within this gene set differ substantially from the ones observed across all the candidate cell wall genes of the study and all the candidate cell wall genes of the initial cell wall QTLs. Specifically, transcription factors (TFs) and genes involved in lignin and hemicellulose metabolism are the categories showing the largest variation. On the one hand, TFs constitute 27% of all the candidate cell wall genes from the 22 selected SQTLs (404 of the 1493 genes) (Figure 5A), a proportion that is 6 and 5 fold higher than what observed among the candidate cell wall genes from the 151 angiosperm genomes (Figure 5C) and from the 610 cell wall QTLs (Figure 5B), respectively. On the other hand, lignin and hemicellulose genes represent 7% (111 genes) and 13% (199 genes) of the cell wall genes from the 22 selected SQTLs, respectively (Figure 5A). These percentages are considerably lower than what observed in the candidate cell wall genes from the 151 genomes (20% for lignin and 25% for hemicellulose) (Figure 5C) and the initial cell wall QTLs (22% for lignin and 26% for hemicellulose) (Figure 5B).

In addition to the proportions above, the types of specific cell wall gene functions that were most represented among the candidate cell wall genes from the 22 selected SQTLs were also evaluated. Regarding TFs, the most represented categories turned out to be *Vascular-related NAC Domain* (*VND*) genes, certain cell wall related *MYB* TFs (*MYB4*, *MYB6*, *MYB7*, *MYB21*, and *MYB32*), *WRKY12*, *NAC Secondary cell wall Thickening* (*NST*), *Ovate Family Protein 4* (*OFP4*), and cell wall related *Ethylene-responsive factors* (*ERF*). All these TFs are either known master regulators of cell wall synthesis in several species, or known regulators of the lignin pathway. Furthermore, some of them are known to display allelic variation associated with significant variation in cell wall properties across different species (see paragraph “The Conservation of Critical Cell Wall Related Loci Through Syntenic Cell Wall Quantitative Trait Loci” and Supplementary Table 9). Concerning lignin genes, our analyses showed that the 22 selected SQTLs contain a relatively high amount of *peroxidases* (*PRX*), *mediator complex subunits* (*MED*), *caffeoyl CoA O-methyltransferases* (*CCoAOMT*) and *caffeoyl shikimate esterases* (*CSE*). The hemicellulose genes from the 22 selected SQTLs displayed instead a relatively large proportion of *mannan synthesis-related* (*MSR*) genes and of different gene families involved in the substitution and remodeling of hemicellulose molecules. These include *BAHD acyltransferases* (*BAHD*), *beta-xylosidases* (*BXL*), *eskimo* genes (*ESK*), *reduced wall acetylation* genes (*RWA*), and *arabinogalactan methylesterases* (*AGM*). Finally, the 22 selected SQTLs harbor also a relatively large amount of genes involved in cell wall remodeling, including *expansin*/*expansin-like* genes (*EXP*/*EXPL*), *extensins* (*EXT*), and *polygalacturonases*/*pectin lyases* (*PG*/*PL*).

**FIGURE 6:**
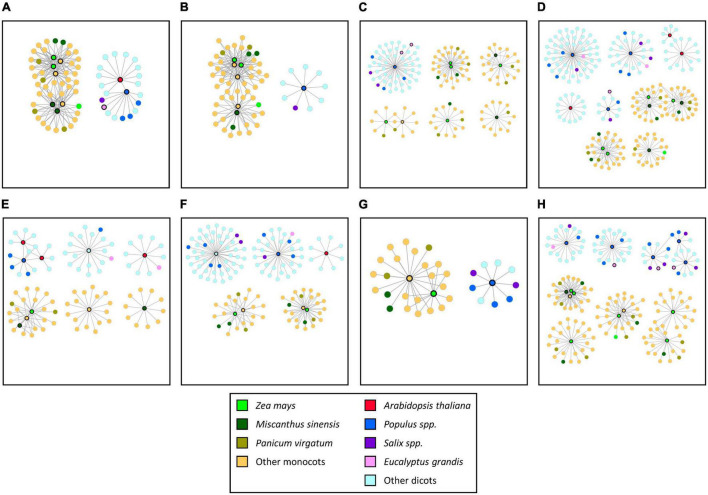
The structure of the syntenic communities of some of the highly conserved cell wall gene families included within 22 SQTLs showing an exceptionally high co-localization of initial cell wall QTLs from diverse plant species and a high extensiveness across angiosperms. Each independent network represents a gene community conserved across a set of species. Network nodes indicate the gene members of syntenic communities from different species (see legend). Network nodes with black bold edges indicate genes contained within initial cell wall QTLs. **(A)** *Vascular-related NAC domain* (*VND*); **(B)** *WRKY12*; **(C)** *NAC Secondary cell wall Thickening factor* (*NST*); **(D)** *caffeoyl shikimate esterases* (*CSE*); **(E) *c****affeoyl CoA O-methyltransferases* (*CCoAOMT*); **(F)** *mediator complex subunits* (*MED*); **(G)** *Reduced Wall Acetylation*; **(H)** *BAHD Acyltransferase*.

The synteny networks of the gene families just discussed were extracted from the 22 selected SQTLs, to analyze the specific patterns of syntenic conservation through SQTLs of all these genes (Figure 6). This analysis showed that the genes above display extensive positional conservation across diverse plant species through SQTLs, as large syntenic communities exist across both monocots and dicots for all these genes (Figure 6 and Supplementary Table 8). Moreover, these communities span diverse species, including important biomass crops, and include several gene members from the initial cell wall QTLs (Figure 6). Interestingly, for several gene families not all the members included in the genomes of the species used for SQTLs detection resulted included in the communities of Figure 6. For example, out of the seven *VND* TFs of arabidopsis, only one member (*AtVND7*) is included in the dicot *VND* syntenic community of Figure 6A. Similarly, of the five *RWA* genes of maize, only one is conserved in the monocot syntenic community of Figure 6G. Overall, these patterns reveal that for several gene families from the 22 selected SQTLs, only a fraction of their members from diverse species are exact positional orthologs of the genes that were originally included in cell wall QTLs. In this sense, the detected SQTLs represent a useful tool to readily identify such positional orthologs, increasing the likelyhood of complete functional gene conservation.

#### The Conservation of Critical Cell Wall Related Loci Through Syntenic Cell Wall Quantitative Trait Loci

**FIGURE 7:**
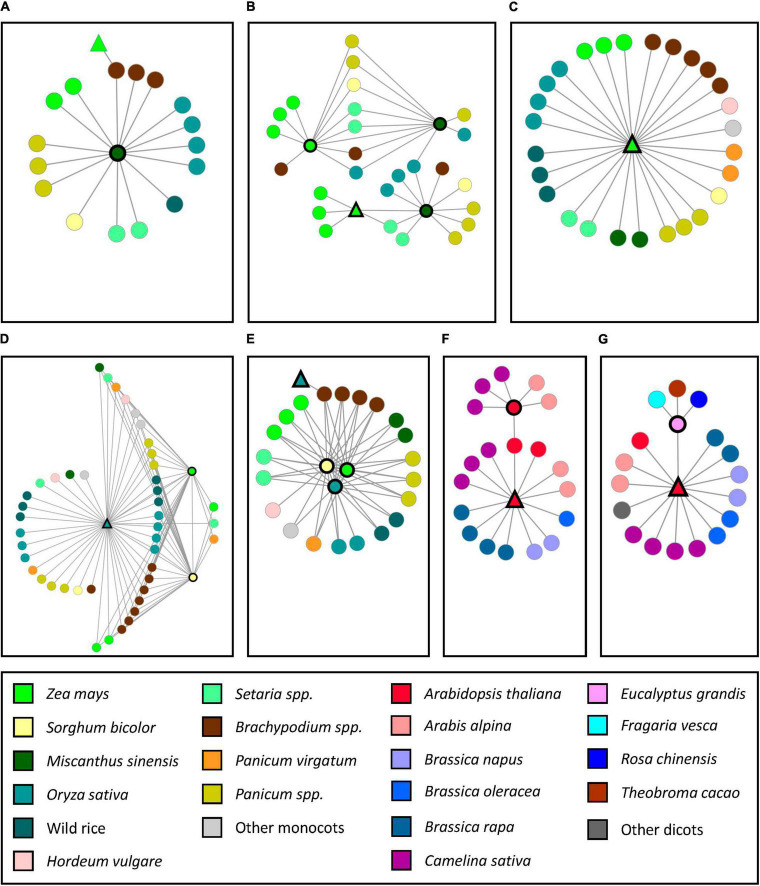
The structure of the syntenic communities of some of the monocot and dicot cell wall genes known to display allelic/mutational variation associated with variation in cell wall quality phenotypes and included in SQTLs. Each independent network represents a gene community conserved across a set of species. Network nodes indicate the gene members of syntenic communities from different species (see legend). Network nodes with black bold edges indicate genes contained within initial cell wall QTLs. Triangular nodes indicate that the gene was shown to display allelic/mutational variation associated with variation in cell wall quality phenotypes. Circular nodes are positional orthologs of those genes. **(A)** *Zea mays Brown Midrib 1* (*ZmBM1*); **(B)** *Zea mays Brown Midrib 3* (*ZmBM3*); **(C)** *Zea mays Brown Midrib 4* (*ZmBM4*); **(D)** *Oryza sativa Brittle culm-like 8* (*OsBCL8*); **(E)** *Oryza sativa Brittle culm-like 9* (*OsBCL9*); **(F)** *Arabidopsis thaliana Cobra-like 4* (*AtCOBL4*); **(G)** *Arabidopsis thaliana Irregular xylem 14* (*AtIRX14*).

Another functional analysis that was performed on the SQTLs encompassed a comparison between all the genes harbored by the 362 SQTLs and a set of 139 cell wall genes from maize, sorghum, rice, arabidopsis and poplar (Supplementary Table 9) that are known from scientific literature to display patterns of allelic/mutational variation with a significant impact on plant cell walls. Since these genes constitute an important set for breeding biomass crops, this analysis aimed at assessing the relevance of SQTLs for crop improvement based on the extent of the conservation of critical cell wall genes through SQTLs. Interestingly, 85% of the genes collected for the grass species (64 out of 75 genes) turned out to be included in SQTLs. Among others, these genes include different *brown-midrib* loci of maize and sorghum (*ZmBM1*, *ZmBM2*, *ZmBM3*, *ZmBM4*, *SbBM2*, and *SbBM3*), 15 of the 17 *brittle culm* and *brittle culm-like* loci of rice, as well as several critical TFs involved in grass cell wall regulation (Supplementary Table 9). In total, the 64 grass genes are involved in 34 different SQTLs. Moreover, the syntenic communities at the basis of these 34 SQTLs revealed that the genomic organization of these genes is extensively conserved across monocot species (Figure 7). In fact, those communities typically span staple crops as maize, rice, barley and sorghum; biomass crops as miscanthus and switchgrass; less utilized relatives of grass cereals as wild rice species, *Panicum miliaceum*, and *Setaria italica*; as well as important species for plant research as *Brachypodium distachyon* (Figure 7). Finally, syntenic communities also revealed interesting differences in the copy number of conserved positional orthologs between species, as well as the occurrence of some of those positional orthologs within initial cell wall QTLs (Figure 7).

For the dicot genes, the situation is opposite, as only 29% of the genes collected for arabidopsis and poplar (19 out of 64 genes) are included in SQTLs (across 11 different SQTLs). Still, the genes contained in SQTLs are relevant, as they include the arabidopsis *IRX14* gene (an essential *xylan synthase* for the synthesis of hemicellulose backbones), an arabidopsis *COBL4* homolog of the sorghum *brittle culm 1* locus, an arabidopsis *CCR*, two poplar *laccases* (*LAC*) and different TFs. Moreover, detailed analyses of gene networks showed that positional conservation through SQTLs is extensive also for these dicot genes (Figures 7F,G). For example, *AtIRX14* turned out to be syntenic through SQTLs to one homolog from Brassicaceae, another one from *Eucalyptus grandis* (included in an initial cell wall QTL), two from *Fragaria vesca*, two from *Rosa chinensis*, and one from *Theobroma cacao* (Figure 7G). In other cases, synteny was restricted to specific plant families, but still extensive within them, as in the case of *AtCOBL4*, which showed extensive conservation through SQTLs across six different species of the Brassicaceae family (Figure 7F).

#### Most and Least Conserved Genes Through Syntenic Cell Wall Quantitative Trait Loci: Overall Patterns

A final functional analysis entailed the evaluation of the degree of conservation through SQTLs of all the candidate cell wall genes included within the 594 initial QTLs spanning at least one gene and used for SQTLs detection. The aim was to identify cell wall gene classes displaying relevant changes in their relative abundance in SQTLs compared to initial QTLs. In this way, we identified 21 functions that can be considered “poorly conserved” (they are much more abundant in QTLs compared to SQTLs) and 27 functions that can be defined “highly conserved” (their abundance displays little or no change in SQTLs compared to initial QTLs, meaning that the majority of their members from initial QTLs are conserved through SQTLs). Table 4 displays these results. Interestingly, out of the 27 highly conserved functions, 15 are different types of TFs that play key roles in the regulation of cell wall quality in plants. These include *Xylem NAC Domain* (*XND*) TFs, different *NST* members, *WRKY12*, *BLH9*, *KNAT3*, *MYB46*/*52*/*54*/*83*/*58*/*63*, *ERF* genes, and *WND* TFs. In addition, *F5H* lignin genes from initial cell wall QTLs are also highly conserved in SQTLs. Finally, other highly conserved genes include different hemicellulose and pectin genes (*PARVUS* and *IRX8*), and the *STELLO* proteins. Concerning the group of “poorly conserved” genes, they include several lignin-related functions (*BGLU*, *COMT*, *DIR*, *G4H*, *CAD*, and *FMT*). Moreover, three main cell wall TFs resulted also included in this group: *E2FC*, *SND2*, and *SND3*.

**TABLE 4:** The cell wall related gene classes from the initial cell wall QTLs whose conservation through SQTLs resulted highest and lowest.

Gene function	Broad cell wall process	Conservation level in SQTLs	Copy number in cell wall QTLs	Copy number in SQTLs (for species with available QTLs)	Copy number decrement SQTLs/QTLs (%)
*STL*	Cellulose synthesis	Highly conserved	1	1	0
*BLH9*	Transcription factor	Highly conserved	4	4	0
*KNAT3*	Transcription factor	Highly conserved	2	2	0
*MYB46*	Transcription factor	Highly conserved	4	4	0
*MYB52*	Transcription factor	Highly conserved	5	5	0
*MYB54*	Transcription factor	Highly conserved	5	5	0
*MYB83*	Transcription factor	Highly conserved	4	4	0
*CESA (II)*	Cellulose synthesis	Highly conserved	10	9	10
*UAfT*	Hemicellulose metabolism	Highly conserved	10	9	10
*UXT*	Hemicellulose metabolism	Highly conserved	10	9	10
*UUAT*	Hemicellulose metabolism	Highly conserved	19	17	11
*NST1*	Transcription factor	Highly conserved	8	7	13
*NST2*	Transcription factor	Highly conserved	8	7	13
*NST3*	Transcription factor	Highly conserved	8	7	13
*WRKY12*	Transcription factor	Highly conserved	8	7	13
*GATL*	Pectin metabolism	Highly conserved	14	12	14
*RHM*	Pectin metabolism	Highly conserved	14	12	14
*XND1*	Transcription factor	Highly conserved	7	6	14
*UGE*	Hemicellulose metabolism	Highly conserved	12	10	17
*MYB58*	Transcription factor	Highly conserved	6	5	17
*MYB63*	Transcription factor	Highly conserved	6	5	17
*UGP*	Cellulose synthesis	Highly conserved	5	4	20
*F5H*	Lignin metabolism	Highly conserved	5	4	20
*WND*	Transcription factor	Highly conserved	39	31	21
*ERF*	Transcription factor	Highly conserved	34	27	21
*IRX8*	Hemicellulose metabolism	Highly conserved	54	42	22
*QUA1*	Pectin metabolism	Highly conserved	54	42	22
*PARVUS*	Hemicellulose metabolism	Highly conserved	44	34	23
*PGI*	Pectin metabolism	Poorly conserved	72	21	71
*FMT*	Lignin metabolism	Poorly conserved	14	4	71
*UGD*	Hemicellulose metabolism	Poorly conserved	4	1	75
*SND2*	Transcription factor	Poorly conserved	4	1	75
*SND3*	Transcription factor	Poorly conserved	4	1	75
*CAD*	Lignin metabolism	Poorly conserved	52	11	79
*C4H*	Lignin metabolism	Poorly conserved	5	1	80
*DIR*	Lignin metabolism	Poorly conserved	44	8	82
*UGT75B1*	Callose synthesis	Poorly conserved	12	2	83
*SPS*	Cellulose synthesis UDP Glu supply	Poorly conserved	6	1	83
*AGAL*	Other cell wall protein	Poorly conserved	6	1	83
*XYN*	Other cell wall protein	Poorly conserved	12	2	83
*COMT*	Lignin metabolism	Poorly conserved	19	3	84
*BGLU45*	Lignin metabolism	Poorly conserved	20	3	85
*KTN1*	Other cell wall protein	Poorly conserved	2	0	100
*MANT*	Other cell wall protein	Poorly conserved	1	0	100
*PNT*	Other cell wall protein	Poorly conserved	1	0	100
*AXY9*	Hemicellulose metabolism	Poorly conserved	1	0	100
*MAN*	Hemicellulose metabolism	Poorly conserved	4	0	100
*E2FC*	Transcription factor	Poorly conserved	2	0	100
*MYB75*	Transcription factor	Poorly conserved	3	0	100

**FIGURE 8:**
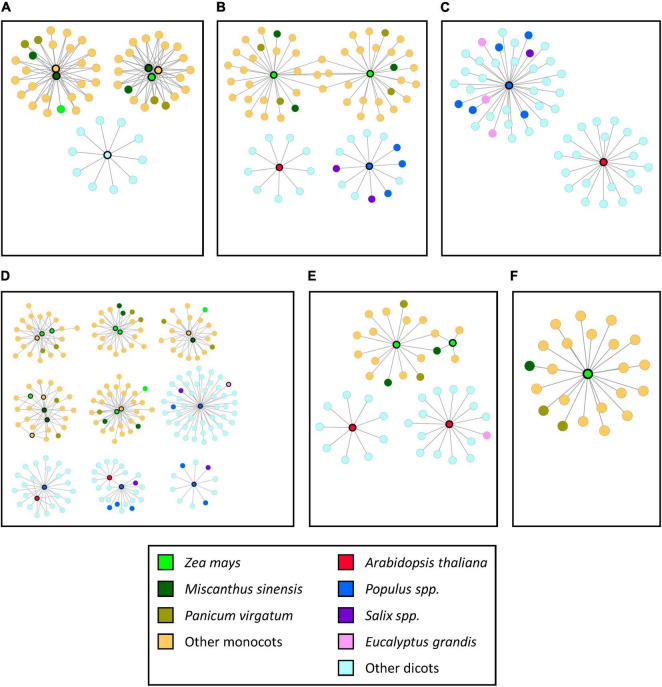
The structure of the syntenic communities of some of the gene families included in initial cell wall QTLs and overall highly conserved through SQTLs. Each independent network represents a gene community conserved across a set of species. Network nodes indicate the gene members of syntenic communities from different species (see legend). Network nodes with black bold edges indicate genes contained within initial cell wall QTLs. **(A)** *Xylem NAC domain* (*XND*); **(B)** *BEL1-like Homeodomain 9* (*BLH9*); **(C)** *Knotted1-like homeobox gene 3* (*KNAT3*); **(D)** *PARVUS*; **(E)** *Ferulate 5-hydroxylase* (*F5H*); **(F)** *STELLO* (*STL*).

The genes that were shown to be highly conserved through SQTLs were also analyzed in terms of genomic patterns of their syntenic conservation through SQTLs. In this sense, their syntenic communities from SQTLs revealed that these genes are extensively positionally conserved across diverse species, both in monocot and dicot plants (Figure 8). Moreover, several members of those syntenic communities are genes that were initially included in cell wall QTLs from different species (Figure 8). Finally, multiple syntenic communities corresponding to different “genomic contexts” in which different members of the gene families above are located were often identified for specific gene groups. For example, the four miscanthus *XND* genes of Figure 8A are grouped into two different syntenic communities. Alternatively, the eight maize copies of the *PARVUS* genes of Figure 8D are divided into five different syntenic communities. This highlights the existence of divergent (conserved) genomic contexts for different gene members of target gene families, which may be revelatory of different evolutionary trajectories or of functional diversification and subfunctionalization within those families. As already mentioned in Section “Cell Wall Genes Within the Syntenic Cell Wall Quantitative Trait Loci With Highest Co-localization of Initial Quantitative Trait Loci,” the distinct genomic contexts for different members of specific gene families highlights once again that SQTLs can be used to identify exact positional orthologs of specific gene copies across a wide range of species.

## Discussion

The major goal of this study was to develop novel tools to translate genomic regions known to control biomass (cell wall) quality from model species to a large set of (orphan) crops. This goal was reached by setting up a strategy for inter-species QTLs projection based on gene synteny, which led to the detection of a large number of SQTLs within different groups of plant species. The detected SQTLs turned out to be extensive across species and to span large portions of the initial cell wall QTLs. Moreover, they highlighted key genes for cell wall quality variation that are positionally conserved across model and orphan species. On the one hand, these results indicate the validity of the approach followed in this research for genomically translating genetic information on traits of interest at a large-scale level and in an effective manner. On the other hand, they also open important considerations on the SQTLs found, with implications for the methodology developed in this research, cell wall genomics, and breeding of (novel) biomass crops. In this section, all these aspects are detailly discussed.

### A Novel Strategy for Translating (Cell Wall) Quantitative Trait Loci Across Diverse Plant Species

A major delivery of this research is the development of a strategy to project QTL regions between plant species by using gene synteny. Specifically, our pipeline detected 362 SQTL regions that span a total of 74 different angiosperm species, starting from a set of 610 cell wall QTLs previously mapped in eight plant species. The detailed analysis of SQTLs properties showed that SQTLs are relatively large in size, are conserved across numerous and diverse plant species (including both monocots and dicots), and overlap with relatively large portions of the initial QTLs used for their detection (see Section “Detection and Descriptive Analysis of Syntenic Cell Wall Quantitative Trait Loci”). All together, these observations indicate that synteny can be successfully used to project QTLs between species in a “translational genomics” framework, and that, because of their high conservation, SQTLs may resemble the functionality of the initial QTLs. In addition, our strategy for SQTL detection – based on network analysis of QTL synteny – is easily scalable to even larger sets of genomes and QTLs, as well as to other traits than the ones used in this study. Therefore, we believe that the approach developed in this research retains high potential for translational genomics also in other contexts than cell wall research.

Nevertheless, since our main focus was on cell wall quality and biomass crops, it is noteworthy that the 74 species included in the detected SQTLs are of high interest for this field of research. In fact, they include important (orphan) biomass crops, like *Miscanthus sinensis*, *Panicum virgatum*, *Eucalyptus grandis*, *Populus trichocarpa*, and *Salix purpurea*; relevant fiber (orphan) species, like different cotton species and *Cannabis sativa*; and some important general bio-based crops for which cell wall quality is relevant for the extraction of bioresources, as *Beta vulgaris*, *Lupinus angustifolius*, *Chenopodium quinoa*, and *Camelina sativa*. Moreover, some vegetable and fruit crops for which cell wall composition is a major determinant of the quality of their food parts are also included in SQTLs, such as *Actinidia chinensis*, *Malus domestica*, *Prunus persica*, and *Solanum lycopersicum*. In all these crops, the regions spanned by SQTLs and the candidate genes therein represent relevant targets for further reverse genetics studies and/or breeding programs. Specifically, some of the species above are currently lacking genetic resources for the improvement of their biomass composition, and the availability of candidate genomic regions and genes from SQTLs could significantly speed up pre-breeding research efforts.

As reported in Section “Detection and Descriptive Analysis of Syntenic Cell Wall Quantitative Trait Loci,” the majority of the SQTLs was detected in the “Poaceae” group of Table 1, which is a notoriously highly syntenic group of plants (Bennetzen and Freeling, 1997). Therefore, the overall synteny of target species on which the initial QTLs are projected appears to be a critical parameter to successfully apply the strategy developed in this study. Moreover, our results highlighted that the overall synteny of the QTLs and of the candidate genes therein is also pivotal to successfully detect SQTLs, and needs to be evaluated beforehand. In the case of cell wall research, the fact that cell wall genes and QTLs turned out to be overall highly syntenic across all angiosperms (Section “Preliminary Analysis of Cell Wall Quantitative Trait Loci and Cell Wall Gene Data”) and that Poaceae species include the majority of biomass crops certainly had a positive influence on SQTLs identification. Nevertheless, our results demonstrated that genomic QTL translation can be successfully achieved also in several groups of eudicot species, such as the ones reported in Table 1. In this case, the level of inter-species synteny appeared more stringent in determining the level of taxonomic distance from species for which initial QTLs were available until which SQTLs identification is possible. Nevertheless, SQTLs were successfully detected across all the eudicot groups of Table 1, highlighting the feasibility of this approach also in these plants. Moreover, the fragmentation of initial QTLs over multiple SQTLs was minimized in smaller groups of eudicots used for SQTLs detection (e.g., Brassicaceae), while SQTLs size was maximized. Furthermore, the fact that cell wall QTLs from poplar and eucalyptus were relatively easily translated across several species (thanks to the high level of synteny found for these species across several eudicot families) is very promising for biomass and cell wall research in eudicots and, specifically, biomass trees. In fact, the improvement of perennial crops such as trees is notoriously time consuming given the long breeding cycles (Clifton-Brown et al., 2018), and translational genomics through genome synteny may therefore significantly speed up pre-breeding research efforts in these species.

To conclude, the analysis of the cell wall gene classes mostly represented within SQTLs also highlighted important considerations on the strategy developed in this study. Specifically, SQTLs turned out to be enriched in cell wall TFs, and several of these TFs belong to the highest layers of cell wall regulation, highlighting the overall relevance of the regions spanned by SQTLs for the regulation of cell wall synthesis, and therefore for controlling variability in cell wall composition. In parallel, the abundant occurrence within SQTLs of genes known to display allelic and mutational variation associated with cell wall composition also indicates the relevance of SQTL regions for controlling cell wall quality. Overall, the general patterns observed in candidate cell wall genes highlight the validity of using gene synteny to project QTLs between species in a meaningful way from the point of view of the genetic architecture of traits.

### Conserved Determinants of Cell Wall Variability as Revealed by Syntenic Cell Wall Quantitative Trait Loci

The functional analyses conducted on SQTLs (Section “Analysis of the Candidate Cell Wall Genes Within Syntenic Cell Wall Quantitative Trait Loci”) highlighted both the presence of critical cell wall candidate genes conserved through SQTLs across large sets of species, as well as classes of genes represented within the initial cell wall QTLs but poorly conserved through SQTLs. On the one hand, the candidate cell wall genes that are highly conserved through SQTLs may represent interesting targets for setting up “universal” approaches to improve biomass crops. Moreover, they also give insights on what can be considered “universal” across sets of plants in terms of the genomic architecture of the trait “cell wall quality.” On the other hand, the combined analysis of conserved and non-conserved candidate cell wall genes through SQTLs open interesting considerations on the most effective mechanisms to manipulate cell wall composition.

A first result of the functional analyses of SQTLs is the consistent occurrence of cell wall related TFs within SQTLs (see Section “Cell Wall Genes Within the Syntenic Cell Wall Quantitative Trait Loci With Highest Co-localization of Initial Quantitative Trait Loci” and “Most and Least Conserved Genes Through Syntenic Cell Wall Quantitative Trait Loci: Overall Patterns”). Since TFs are important players in the regulation of plant traits, including plant cell wall (Rao and Dixon, 2018; Zhang et al., 2018), and are often causative genes at the basis of QTLs (Barrière et al., 2012; Courtial et al., 2014), their consistent conservation through SQTLs highlights the value of these tools to pinpoint relevant conserved candidate genes across diverse species. The analysis of the cell wall TFs included in SQTLs reveled that they include master regulators of cell wall biosynthesis, such as the *VND* and *NAC* TFs. These TFs regulate the global deposition of secondary cell walls in plant vessels and fibers, respectively, and are able to bind to critical structural genes at the basis of cellulose, xylan and lignin biosynthesis (Taylor-Teeples et al., 2015; Zhong and Ye, 2015). In addition, their functionality is hypothesized to be largely conserved across diverse plant species (Nakano et al., 2015; Zhong and Ye, 2015), and genetic modifications of these genes resulted in plant phenotypes with altered cell wall composition and quality, including improvement of biomass saccharification (Iwase et al., 2009; Yoshida et al., 2013). All together, this evidence highlights the relevance of *VND* and *NAC* TFs for the improvement of cell wall composition in plants. In this context, SQTLs can be used to readily detect sets of *VND* and *NST* orthologs located on conserved genomic contexts across species. In addition, SQTLs could also be used to discriminate between the different copies of these genes when mining the exact positional orthologs of specific gene members with a higher functional relevance, to maximize the likelihood of a complete gene functional conservation (Dewey, 2011). For example, it has been demonstrated that out of the seven *VND* copies of Arabidopsis, one – *AtVND7* – is the major player of the *AtVND* family, by acting as the transcriptional terminus of these genes and by impacting cell wall deposition the most (Yamaguchi et al., 2011; Endo et al., 2015). Interestingly, *AtVND7* is the *AtVND* member that resulted conserved through SQTLs (red dot circled with bold border in Figure 6A). The other dicot genes of Figure 6A syntenically connected to *AtVND7* represent therefore the exact positional orthologs of *AtVND7* – and not of the other *AtVND* copies – in other species. Therefore, SQTLs can discriminate between the different members of critical gene families when defining gene targets for plant research.

In addition to *VND* and *NAC* TFs, *WRKY12* is another master regulator of cell wall biosynthesis that acts as repressor of lignin deposition (Wang et al., 2010) and that was largely conserved through SQTLs across both monocots and dicots. Mutations at *WRKY12* heavily affect the relative content of lignin, cellulose, and hemicellulose, as well as the production of total stem biomass in Arabidopsis (Wang et al., 2010). Moreover, a *WRKY12* gene from *Miscanthus lutarioriparius* was shown to promote flowering when inserted in Arabidopsis (Yu et al., 2013). This information highlights once again that SQTLs can pinpoint relevant genes for improving biomass crops that thanks to syntenic conservation can be easily mapped across the different species of the study. Moreover, because of its properties, WRKY12 could specifically represent an attractive target for the parallel modification of cell wall quality, biomass production, and flowering time.

Other TFs that were highly conserved through SQTLs include *OFP4*, *ERFs*, *BLH9*, *KNAT3* and several *MYB* genes. These genes are all important regulators of cell wall deposition across several species, even if their role is less central than the one of the *VND*, *NST*, and *WRKY12* TFs (Zhong and Ye, 2015). Nevertheless, their functional redundancy with other TFs (Taylor-Teeples et al., 2015) may underly a higher chance of finding useful allelic variation at the loci coding for these genes in target crops, as the selection pressure exerted on these genes might have been relatively relaxed. For all these genes, SQTLs may again be used to readily identify homologs laying in conserved genomic contexts across the species of this study, including monocot and dicot biomass species (miscanthus, switchgrass, poplar, willow) across which the genomic organization of these genes appeared extensively conserved. In species of interest, allelic variation at the loci mapped through SQTLs may then be studied with novel methodologies for targeted sequencing (Scaglione et al., 2019), eventually leading to the detection of favorable germplasm sources to be used in breeding programs.

In addition to TFs, SQTLs contained a large amount of genes involved in the substitution and/or remodeling of cell wall polymers. This class of genes is also of high importance for the improvement of biomass crops, as both the degree of substitution of cell wall polymers with a variety of chemical moieties and the re-building of cell wall polymers during cell wall metabolism are pivotal processes to determine the amenity of plant cell walls to deconstruction (Van Der Weijde et al., 2013; Torres et al., 2015). In this perspective, SQTLs may be used to identify critical candidate genes in crops with scarce genetic resources thanks to positional orthology with key genes included in cell wall QTLs in model species. Specifically, some of the genes involved in the remodeling of cell wall polymers and conserved through SQTLs are typically found in single or low copy-number in plant genomes, including *PARVUS* and *ESK*. Both these genes contribute to xylans synthesis, with *PARVUS* being likely involved in the synthesis of the xylans reducing ends (which in turn are presumably primers for total xylan synthesis) (York and O’neill, 2008), and *ESK* being involved in xylans mono-acetylation (Yuan et al., 2013). Even if the precise functioning of these genes is far from being understood (Smith et al., 2017), the traits on which they are presumably involved – xylans amount and xylans substitutions – are preeminent targets for improvement of biomass crops (Van Der Weijde et al., 2013; Torres et al., 2015). Therefore, the members of the *PARVUS* and *ESK* gene families retained in SQTLs could certainly represent interesting targets for further reverse genetic studies, also because of the presence of some of their members within the initial cell wall QTLs used for SQTLs detection. In contrast to *PARVUS* and *ESK*, other genes involved in the remodeling of cell wall molecules and highly conserved through SQTLs belong to large families, like the *BAHD*, *BXL*, *RWA*, *EXT*, and *PG*/*PL* genes. These genes perform different functions within plant cell walls [see Zhong et al. (2019) for a review], but have all been indicated as candidate genes for modifying biomass quality by changing the content and biochemical properties of cell wall molecules (Bartley et al., 2013; Biswal et al., 2014; Pawar et al., 2017). Interestingly, for several of these gene families from model species it has been shown that different members can perform different functions, or can exert their functions in different plant organs or developmental stages (Nakhamchik et al., 2004; Tuominen et al., 2011; Cao, 2012). In this sense, as previously discussed for *VND* TFs, SQTLs may again both allow the quick identification of conserved positional orthologs across diverse species and help to discriminate between multiple family members to decide which copies to target in plant research or plant breeding.

To conclude, in addition to the gene classes mentioned so far, the analysis of SQTLs highlighted the presence of gene families that, despite being represented in the initial cell wall QTLs used for SQTLs detection, revealed to be poorly conserved through SQTLs (Section “Most and Least Conserved Genes Through Syntenic Cell Wall Quantitative Trait Loci: Overall Patterns”). Interestingly, several lignin structural genes involved in the pathway leading to monolignol synthesis were included in this category. This observation, together with the fact that several lignin TFs were instead found highly conserved through SQTLs, suggests that targeting TFs may be a more successful and more interapplicable strategy for modifying lignin across different species compared to the targeting of lignin structural genes. In this context, the *ferulate 5-hydroxylase* gene (*F5H*) represents an exception, as it was found highly conserved through SQTLs (Figure 8). The genetic manipulation of this gene is known to alter monolignol ratios and can substantially improve biomass saccharification in several species (Stewart et al., 2009; Weng et al., 2010). Therefore, the copies included in SQTLs may represent interesting breeding targets. In addition, the extensive positional conservation of *F5H* across several monocot and dicot species suggests that targeting this gene may represent a “universal” approach to biomass improvement.

## Conclusion

The present study is the first research, to our knowledge, to develop a successful strategy to project a set of (cell wall) QTLs across a large set of species in a translational genomics framework and through the use of gene synteny. The approach developed in this study represents a novel tool to assist breeding of (orphan) lignocellulosic biomass crops, and can potentially be applied also to other sets of species and traits than the ones used here. The functional analysis of SQTLs demonstrated that those regions retain conserved critical genes for cell wall quality – as *VND*, *NAC*, and *WRKY12* transcription factors, *PARVUS*, *RWA*, or *ESK* genes involved in cell wall remodeling, and several *F5H* copies – which could represent targets for “universal” approaches for biomass improvement. In this sense, future research efforts may be directed to evaluate the allelic variation of SQTL regions across diverse species and to further validate the relevance of the candidate genes found through reverse genetics.

## Data Availability Statement

The original contributions presented in the study are included in the article/Supplementary Material, further inquiries can be directed to the corresponding author/s.

## Author Contributions

FP designed and conducted the research and wrote the manuscript, with inputs and supervision from EL and LT. DV and HR co-worked with FP in designing and performing the detection of syntenic cell wall QTLs. All authors corrected and approved the final manuscript.

## Conflict of Interest

The authors declare that the research was conducted in the absence of any commercial or financial relationships that could be construed as a potential conflict of interest.

## Publisher’s Note

All claims expressed in this article are solely those of the authors and do not necessarily represent those of their affiliated organizations, or those of the publisher, the editors and the reviewers. Any product that may be evaluated in this article, or claim that may be made by its manufacturer, is not guaranteed or endorsed by the publisher.

## Supplementary Material

Supplementary Figure 1 The distributions of synteny levels (as percentage of genes of certain species syntenic with all the other genes from all the other species) for relevant species/families inspected for determining plant groups to be used for SQTLs detection (Table 1).
