# Supplementary figures and images for: Detection and Analysis of Syntenic Quantitative Trait Loci Controlling Cell Wall Quality in Angiosperms
Source: Front Plant Sci. 2022 Mar 3;13:855093. doi: 10.3389/fpls.2022.855093 (PMC8928447; doi:10.3389/fpls.2022.855093)

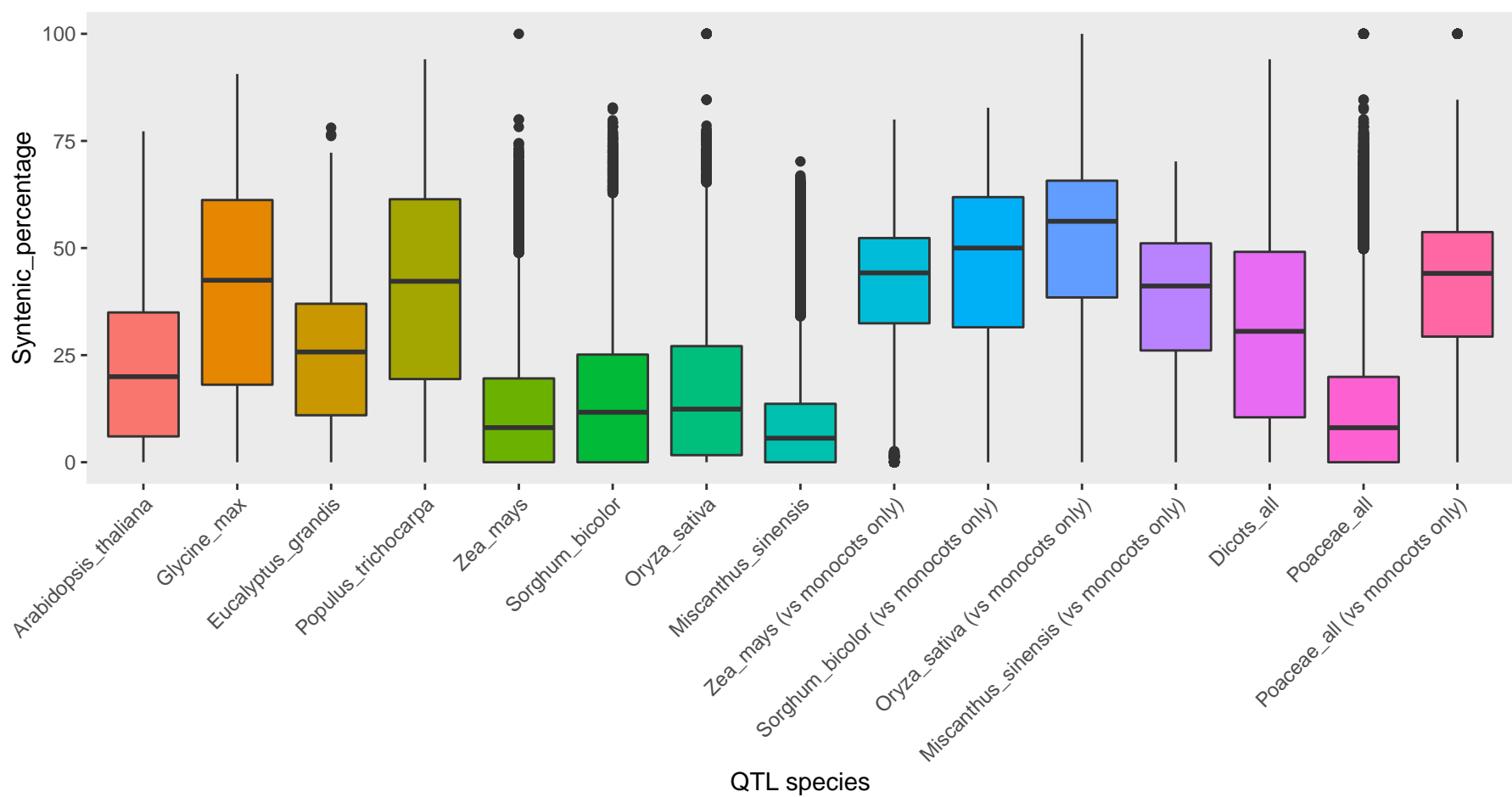

Supplement: Supplementary Figure 1 — The distributions of synteny levels (as percentage of genes of certain species syntenic with all the other genes from all the other species) for relevant species/families inspected for determining plant groups to be used for SQTLs detection (Table 1). [file Image_1.pdf]
